# Therapeutic bacteria to combat cancer; current advances, challenges, and opportunities

**DOI:** 10.1002/cam4.2148

**Published:** 2019-04-05

**Authors:** Mansour Sedighi, Abed Zahedi Bialvaei, Michael R. Hamblin, Elnaz Ohadi, Arezoo Asadi, Masoumeh Halajzadeh, Vahid Lohrasbi, Nima Mohammadzadeh, Taghi Amiriani, Marcela Krutova, Abolfazl Amini, Ebrahim Kouhsari

**Affiliations:** ^1^ Department of Microbiology, School of Medicine Iran University of Medical Sciences Tehran Iran; ^2^ Wellman Center for Photomedicine Massachusetts General Hospital Boston Massachusetts; ^3^ Department of Dermatology Harvard Medical School Boston Massachusetts; ^4^ Harvard‐MIT Division of Health Sciences and Technology Cambridge Massachusetts; ^5^ Golestan Research Center of Gastroenterology and Hepatology Golestan University of Medical Sciences Gorgan Iran; ^6^ 2nd Faculty of Medicine, Department of Medical Microbiology Charles University and Motol University Hospital Prague Czech Republic; ^7^ Laboratory Sciences Research Center Golestan University of Medical Sciences Gorgan Iran

**Keywords:** bacteriotherapy, cancer, chemotherapy, immunotherapy, radiotherapy, tumoricidal agents

## Abstract

Successful treatment of cancer remains a challenge, due to the unique pathophysiology of solid tumors, and the predictable emergence of resistance. Traditional methods for cancer therapy including radiotherapy, chemotherapy, and immunotherapy all have their own limitations. A novel approach is bacteriotherapy, either used alone, or in combination with conventional methods, has shown a positive effect on regression of tumors and inhibition of metastasis. Bacteria‐assisted tumor‐targeted therapy used as therapeutic/gene/drug delivery vehicles has great promise in the treatment of tumors. The use of bacteria only, or in combination with conventional methods was found to be effective in some experimental models of cancer (tumor regression and increased survival rate). In this article, we reviewed the major advantages, challenges, and prospective directions for combinations of bacteria with conventional methods for tumor therapy.

## 1 INTRODUCTION

Cancer remains one of the most common causes of death throughout the world.^1^, ^2^, ^3^ In 2018, the new cases and deaths of cancer were reported 18.1 and 9.6 million, respectively.^4^ By 2030, it is expected that there will be ~17 million deaths.^1^ These statistics emphasize the urgency of finding novel and more effective treatments. The historical treatment options for cancer, including surgery, radiotherapy, chemotherapy, and immunotherapy all have some limitations. Treatment of various cancers is difficult due to the multifactorial physiology (including problems with volume, site, stage, and metastasis of the tumor). Moreover, resistance often emerges to reduce the initial effectiveness of chemotherapy, radiotherapy, and immunotherapy, leading to poor tumor control, and many side effects occur during or after the treatment).^5^ Alternative or complementary therapies such as gene therapy, diet therapy, photodynamic therapy, insulin potentiating therapy, HAMLET (human alpha‐lactalbumin made lethal to tumor cells), telomerase therapy, hyperthermia therapy, dichloroacetate, non‐invasive RF cancer treatment, and bacteriotherapy have been proposed to improve and increase the effectiveness of conventional cancer therapy.^6^

The use of therapeutic bacteria is one approach that may be able to overcome some of the limitations of conventional cancer therapy as stated above. Bacteria alone can act as potent antitumor agents. Another remarkable feature of bacteria is their ability to be genetically engineered to alter their ability to synthesize and release specific compounds, and tailor their metabolic pathways. Publications on the use of bacterial anti‐cancer therapies have grown significantly over the past few decades. Therapeutic bacteria can especially target the hypoxic areas of tumors and actively penetrate the tissue, and can allow different strategies such as the secretion of toxins/enzymes including proteases and lipases to be tested. Bacteria can be used as vectors to carry tumoricidal agents and immunotherapeutic agents, thereby destroying tumor cells (Table 1)*.* 7, 8, 9, 10 However, the fight against cancer is not expected to be won any time soon, and so creative efforts to harness the power of bacteria for cancer treatment will still continue. This review covers the use of bacteria as anticancer agents to improve cancer treatment.

**Table 1:** The treatment strategy of bacteria in cancer therapy

Treatment strategy	Type of bacteria, treatment approach	Outcome	Ref.
Immunotherapeutic agents	*Streptococcus pyogenes*, intentionally infected a cancer patient with erysipelas	Rapid tumor regression	149
	*Streptococcus pyogenes*, intentionally infected a cancer patient with erysipelas	Regression of cancer	150
	Bacillus Calmette‐Guerin, injection into patients with tuberculosis	Reduced frequency of cancer	151
	*Clostridium* spp, concurrently suffered from gas gangrene in patients with tumor	Tumor regression	152
	Attenuated *Salmonella typhimurium*, vaccination of the B16F10 tumor‐bearing mice by derivatives *Salmonella typhimurium* (SL1344 InvA or SL3261AT InvA	An antitumor effect	51
	*Listeria monocytogenes,* vaccination a recombinant *Listeria monocytogenes* (Lm‐NP) on breast, melanoma, and cervical cancer	Regression growth all types of tumors	153
Vectors/spores to carry tumoricidal agents	*Clostridium acetobutylicum* DSM792, cloned of the construction (pIMP1eglArIL2) of the rIL2 expression/secretion vector into *Clostridium acetobutylicum* DSM792	A significant increase in secretory production of biologically active rat interleukin‐2	154
	*Clostridium novyi*‐NT, IV injection of *C novyi*‐NT spores and a single IV dose of liposomal doxorubicin (Doxil) administered into mice bearing colorectal cancer	Elimination of tumors	126
	*C novyi‐NT and C. sporogenes,* conjugation of pMTL‐555‐VHH construct of a VHH‐AG2 expressing vector (an anti HIF‐1α) into these bacteria	Rise of delivery of therapeutics agents	44
	*C novyi‐NT, IV* administration of HTI‐286, docetaxel, vinorelbime, and MAC‐321 in combination with or without *C novyi‐NT* spores into mice bearing HTC 116 xenografts	Hemorrhagic necrosis of tumors	155
	*C novyi*‐NT spores, IV injection *C novyi*‐NT spores into CT26 and RENCA tumors in mice and VX2 tumor in rabbits	Relatively treated in mice and rabbits with cancer	156
	*Bifidobacterium longum* 105‐A and 108‐A, IV injection of the pBLES100 (constructed by cloning a *B longum* plasmid and a gene encoding spectinomycin adenyltransferase AAD from *Enterococcus faecalis* into the *E coli* vector pBR322) to B16‐F10 melanoma tumor‐bearing mice	Increase in specific gene delivery vectors in the tumor	74
Bacterial toxins/enzymes	*Salmonella enterica Serovar Typhimurium,* oral administrated construction of* *Salmonella‐based survivin vaccine into BALB/c, colon, DBT, and GL261 glioblastoma ‐bearing mice	Vaccine as an adjuvant against different types of cancer	157
	*Streptococci* and *Serratia marcescens*, injection of bacterial concoction derived from heat‐killed streptococcal and *Serratia marcescens* (Coley's Toxin) into body, sarcomas	A severe erysipelas infection led to the cure of cancer	158
	*E coli* BM2*‐1* strain, direct inoculum of Cytotoxic Necrotizing Factor‐1 (CNF‐1) to the HEp‐2 cells (exposed to UVB irradiation)	Activation of the Rho GTP‐binding protein and prevent apoptosis in epithelial cells	159
	*Corynebacterium diphtheriae*, the incubation of the Vero cells for 1 h in growth medium with different amounts of nicked^124^ I‐labeled diphtheria toxin (DT)	Inhibition of protein synthesis and subsequent cell lysis and/or induction of apoptosis Vero cells	160
	*Clostridium perfringens,* intratumoral injections of either 2, 10 µg of *Clostridium perfringens* enterotoxin (CPE) in *xenografts of T47D breast cancer cells in mice*	Rapid and dose‐dependent cytolysis	161
	*Clostridium botulinum,* administration of botulinum neurotoxin (BoNTs) into tumors	BoNTs an effect on the tumor microenvironment and more effective destruction of radiotherapy and chemotherapy in cancer cells	129
	*Pseudomonas aeruginosa*, IV injection of the chimeric fusion protein interleukin‐4‐Pseudomonas exotoxin (IL4‐PE) into GBM induce in nude mice and, intratumor administration of IL4‐PE in malignant astrocytoma in a phase I clinical trial	A significant antitumor activity	162

## 2 BACTERIA IN CANCER THERAPY

**Figure 1:**
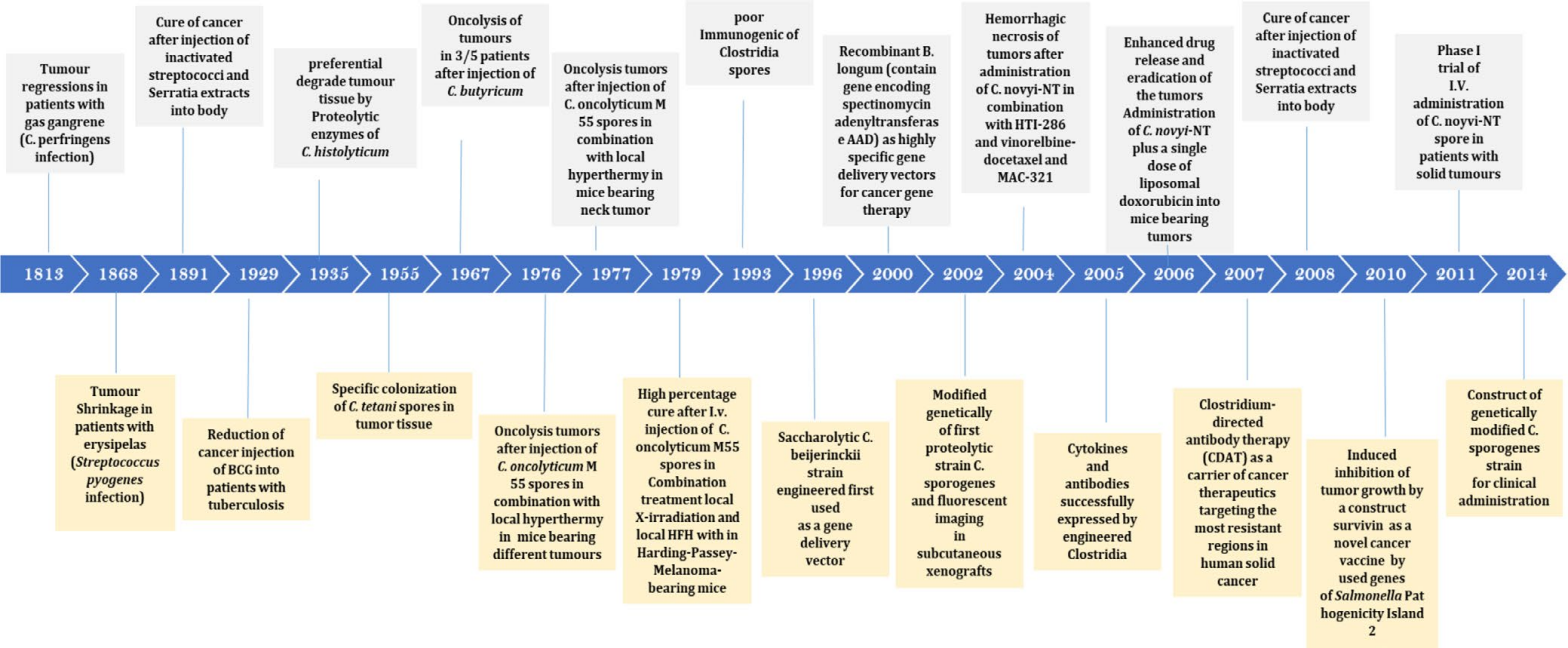
Timeline | The history uses of therapeutic bacteria in oncology

**Figure 2:**
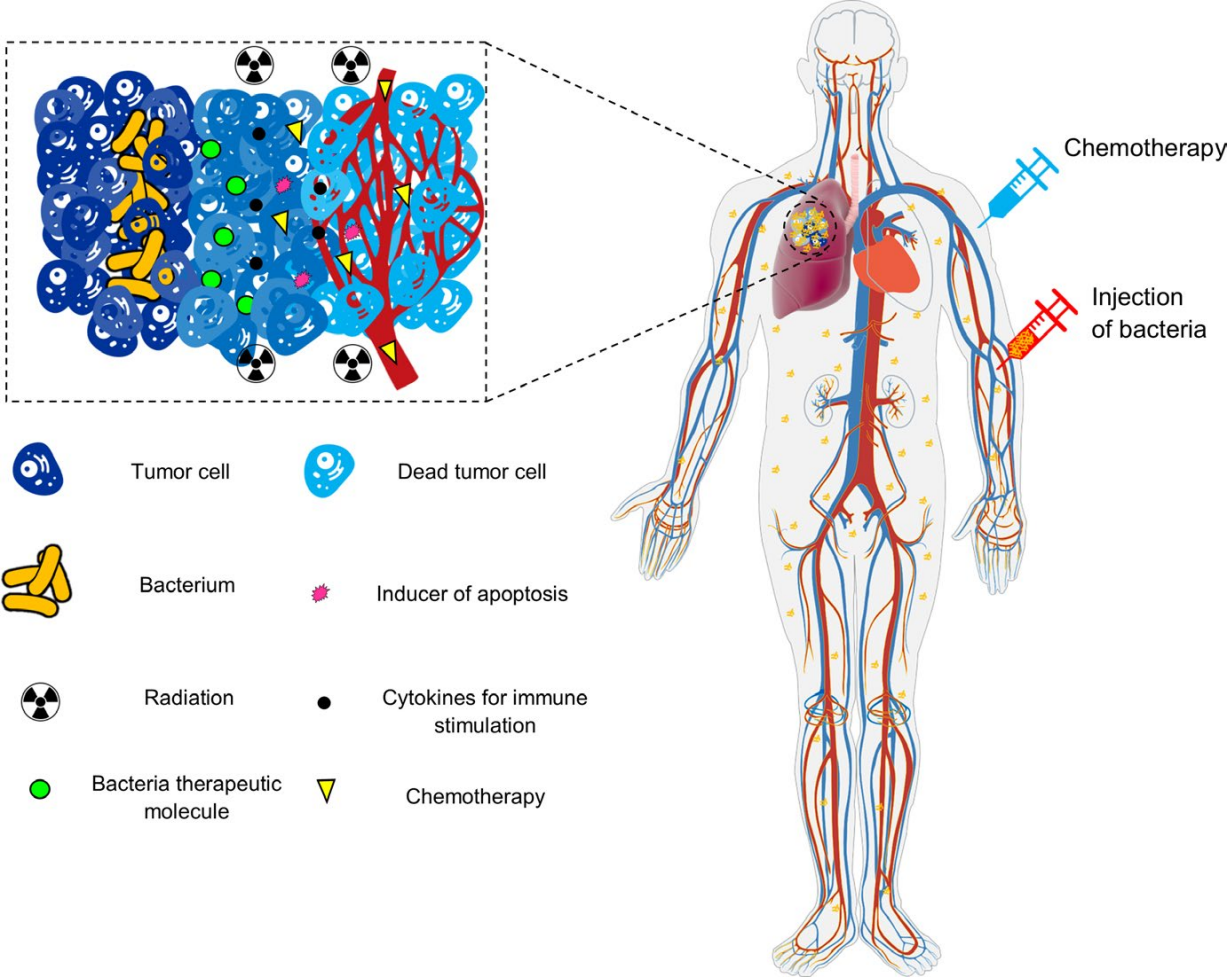
Schematic of therapeutic bacteria strategies against hypoxic tumor adapted from Forbes^7^

Cancer is a challenging disease, which requires a multi‐pronged approach for effective treatment.^11^ The historical role of bacteria as anticancer agents was recognized as long as one century ago (Figure 1). For the first time clinicians used live bacteria (*Streptococci* and *Clostridia*) for cancer treatment. Today, genetically modified bacteria are mostly used for this purpose.^12^, ^13^, ^14^, ^15^, ^16^ Bacteria can be used in cancer therapy by taking advantage of different strategies (Figure 2). These strategies include native bacterial toxicity, combination with other therapies, bacteria that can control expression of anticancer agents, expression of tumor‐specific antigens, gene transfer, RNA interference, and pro‐drug cleavage.^7^ The use of whole live, attenuated and/or genetically modified bacteria alone, or in combination with conventional agents has been tested in several experimental models of cancer. The most common bacteria used in this field are the genera *Salmonella*, *Clostridium*, *Bifidobacterium*, *Lactobacillus*, *Escherichia*, *Pseudomonas*, *Caulobacter*, *Listeria*, *Proteus,* and *Streptococcus*.^8^, ^17^, ^18^, ^19^, ^20^ The use of three species of bacteria, *Clostridia*, *Bifidobacteria*, and *Salmonellae* as vectors for delivering or expressing tumor suppressor genes, anti‐angiogenic genes, suicide genes, or tumor‐associated antigens has been tested in animal models bearing various tumors.^8^, ^21^, ^22^, ^23^, ^24^ Some clinical trials have already been conducted displaying partial responses, and thus further investigation should be performed in humans.^8^ Also, modified bacteria can be used for theranostic applications, since they can be detected using magnetic resonance imaging (MRI) or positron emission tomography (PET) as dual therapeutic and diagnostic agents.^7^, ^25^, ^26^, ^27^, ^28^, ^29^, ^30^, ^31^

## 3 GENETICALLY MODIFIED BACTERIA IN CANCER THERAPY

Gene therapy is a forward‐looking alternative approach to cancer therapy. Selective targeting and destruction of tumor cells are the major advantages of gene therapy.^8^, ^12^, ^32^, ^33^ Genetically modified bacteria may also be able to lower pathogenicity to the host and increase the antitumor efficacy.^32^, ^34^ Recently, a number of studies have developed a new approach for cancer therapy using genetically engineered bacteria designed to express reporter genes, cytotoxic protein and/or anticancer agents, and tumor‐specific antigens.^10^, ^34^, ^35^ It has been found that genetically modified bacteria can have a more significant multiplication in tumors than in normal tissues.^36^, ^37^ The ability of *Salmonella typhimurium* serovar VNP20009 and *Clostridium butyricum* M55 to selectively colonize tumors has allowed them to be used as delivery vectors in mouse tumor models, without severe immune responses or toxic side effects. However, the results of some of these studies were less promising than expected.^8^, ^18^, ^19^, ^38^, ^39^, ^40^ Nevertheless, *Clostridia* strains (*C acetobutylicum* and *C beijerinckii)* can be successfully engineered to express genes encoding specific bacterial enzymes (cytosine deaminase, nitroreductase), or murine tumor necrosis factor alpha (m‐TNFα), producing more promising antitumor effects.^41^, ^42^, ^43^ A number of studies found that bacteria were able to produce antibodies that could bind to hypoxia inducible factor 1α (as a crucial transcription factor with a role in tumor development).^7^, ^44^, ^45^ Clinical trials have demonstrated that the engineered *S typhimurium* and *Clostridium novyi* ‐NT expressing HlyE or Stx2 (an acidic pH‐responsive promoter) or recA (a 38 kDa protein essential for the repair and maintenance of DNA) activated the host immune system to express cytokines such as interleukin‐2 (IL‐2), IL‐4, IL‐18, CC chemokine‐21, and consequently led to the regression and necrosis of tumors**.** 8, 46, 47, 48 These studies suggested that a combination of bacteriotherapy with radiotherapy, immunotherapy, or chemotherapy could be a novel and useful approach to cancer treatment.

## 4 BACTERIA AS IMMUNOTHERAPEUTIC AGENTS IN CANCER THERAPY

Cancer immunotherapy involves triggering a specific immune response in patients to allow various kinds of host immune cells to attack the cancer cells. It is believed that once the activated host immune cells (mainly tumor antigen‐specific CD8+ and CD4 +T lymphocytes that have been activated and stimulated) can recognize and destroy tumor cells. Bacterial infections (such as those caused by *C novyi*) can lead to the production of heat shock proteins such as Hsp70 which is released from necrotic cells, and pathogen‐associated molecular patterns (PAMPs) which are released from bacteria.^49^ Hsp70 causes maturation of dendritic cells, which are the professional antigen‐presenting cells required for the generation of effective antigen‐specific immune responses. PAMPs bind to and activate toll‐like receptors, stimulating up‐regulation of pro‐inflammatory cytokines (eg, IL‐12), and costimulatory molecules (eg, CD40). Subsequently, these mediators cause production of interferon gamma (IFN‐γ) and a Th1‐dependent cell‐mediated response will commence, essentially mediated by CD8+ effector cells.^50^ CD8+ lymphocytes isolated from *C novyi NT*‐treated mice can in turn stimulate acquired immunity in a tumor‐specific model. Avogadri et al, have described an interesting approach to melanoma immunotherapy dependent on the ability of intracellular bacteria like *S typhimurium* to infect host cells.^51^ They observed that *S typhimurium* used a type‐3 secretion system (T3SS) to infect tumor cells. Essentially, mutant strains, which were defective in T3SS lost the ability to enter tumor cells, both in vivo and in vitro. Tumor cells infected by *Salmonella* are not directly destroyed by the *S typhimurium*; but rather bacterial antigens are presented and become targets for anti‐*Salmonella*—specific T cells, a process that has not been fully explained. Furumoto et al, have reported that compounds derived from bacteria (CpG oligonucleotides) can be used to activate dendritic cells and cause complete regression of B16F10 melanoma tumors, which are known to be highly immunosuppressive in mice.^52^

Recombinant *Escherichia coli* strains have been utilized for over 30 years as a standard tool in molecular biology, and are broadly used for manufacturing recombinant proteins. These strains can also be used for the delivery of tumor antigens into dendritic cells. The simultaneous production of listeriolysin O (LLO; a pore‐forming cytolysin released from *Listeria monocytogenes*) together with ovalbumin (OVA; a model tumor antigen) in *E coli* strains led to the MHC class I presentation of the OVA Kb‐restricted epitope, SIINFEKL, after phagocytosis of the microorganisms by macrophages.^53^ LLO is expressed as a bacterial cytoplasmic protein, and is only released subsequent to the uptake of the *L monocytogenes* by phagocytosis and degradation within the phagocytic vesicles. LLO then punctures the phagosome, permitting its release into the cytosol together with co‐expressed proteins, for processing and presentation by the MHC class I pathway. A recent study showed that, if applied several times, a combination of PAMPs could eradicate solid tumors in cancer bearing mice.^54^

## 5 BACTERIAL TOXINS OR ENZYMES IN CANCER THERAPY

Several pathogenic microorganisms express and release particular protein toxins that serve to suppress the immune response of the infected host. Some of these have been tested to some extent for cancer therapy.^55^ Commonly, they catalyze the covalent alteration of specific proteins. In that way, they can inhibit the production or release of antibodies and cytokines. Moreover, they can inhibit macrophage migration and disrupt the barrier function of epithelial cells. Often, these toxins are powerful enzymes with high specificity against their cellular substrates, which are frequently signaling molecules. These enzymatic toxins have the capability to alter their substrates in the cytosol after bacteria enter the cells. A few toxins are able to change the function and morphology of the cells, or possibly kill the host cells. Since many of these toxins have well‐known structures, cellular receptors, molecular mechanisms, and uptake pathways, they have been utilized to analyze or influence particular signaling pathways of mammalian cells.^55^ Bacterial toxins are the most powerful cytotoxins produced by bacteria themselves. Cytolysin A (ClyA; also known as HlyE) is a bacterial enzyme toxin, which works by making pores in eukaryotic cell membranes and triggering caspase‐mediated cell death.^10^, ^47^ A few studies have found that treating mice with *S typhimurium* or *E coli* strains expressing the ClyA toxin inhibited tumor growth.^10^, ^47^ Three of the cytotoxins have been found to belong to the TNFα superfamily: TNF‐related apoptosis‐inducing ligand (TRAI‐l), FAS ligand (FAS‐l), and TNFΑ.^7^, ^56^ These proteins selectively cause programmed cell death via death receptor pathways, activating the apoptotic mediators, caspase 3 and caspase 8.^56^ Recently, in a report of “photo‐controlled bacterial metabolite therapy”, Zheng et al^57^ developed a biotic/abiotic hybrid system. They combined carbon nitride (C_3_N_4_) with an *E coli* strain that was able to produce nitric oxide (NO). In a mouse model, the C_3_N_4_ loaded bacteria were accumulated throughout the tumor, and the treatment resulted in a significant antitumor activity (~80% inhibition of tumor growth). Furthermore, cell cycle inhibitors (e.g. cytolethal distending toxins, CDTs, and the cycle inhibiting factor, Cif) blocked cell division and were proposed to compromise the immune system by impeding the clonal expansion of lymphocytes. Conversely, cell cycle stimulators, for example, the cytotoxic necrotizing factor (CNF) stimulate cell proliferation and interfere with cell differentiation.^58^, ^59^ Bacterial toxins that block or stimulate the eukaryotic cell cycle have been called “cyclomodulins”. For instance, CNF is a cell‐cycle stimulator released by some bacteria such as *E coli*. CNF stimulates the G1‐S cycle transition and increases replication of DNA. However, the overall number of cells does not increase, and the cells become multinucleated instead, possibly through the ability of toxin to prevent cell differentiation and trigger cell apoptosis.^60^ Cif is involved in enterohemorrhagic *E coli* and enteropathogenic *E coli*, while CDTs are produced by several Gram‐negative bacterial species, and *Salmonella typhi* and *Campylobacter jejuni*. The antitumor effects of bacterial toxins could be associated with reduced side effects compared to traditional antitumor therapy. These bacterial toxins could be combined with anti‐cancer drugs, or with irradiation to enhance the efficacy of cancer therapy.^61^

## 6 BACTERIAL SPORES AND VECTORS AS TUMORICIDAL AGENTS

Live bacterial vectors may be valuable tools for the development of new cancer therapies, which can be added to the collection of existing drugs.^62^ In one aspect of this novel methodology, bacteria are modified to deprive cancerous cells of oxygen, thereby causing tumor death.

The interest in utilizing bacteria as anticancer agents goes back to the end of the 19th century; however, with the advent of molecular biology, this methodology has also been recently revisited.^63^, ^64^ Over the past 50 years, many strains of obligate and facultative anerobic bacteria have been investigated as oncolytic agents owing to their ability to specifically multiply in oxygen‐poor (hypoxic) tissues.^65^, ^66^, ^67^ Early results suggested that anerobic bacteria particularly targeted solid tumors, triggering an inflammatory response inside the tumor followed by tumor regression in up to 30% of cases for instance after administration of bacterial spores.^68^, ^69^, ^70^ Several strains of *Bifidobacteria*, *Salmonella* sp,^71^ *E coli*,^72^, ^73^ and *Clostridium* 74 have been shown to selectively colonize the hypoxic areas of tumors and destroy tumor cells, thereby providing an additional specific tumor‐targeted therapy.^62^ Several studies have suggested that bacteria have been engineered to express a cytotoxic protein or reporter gene could be utilized in tumor treatment or tumor imaging.^75^ These genetically modified bacteria multiply in tumors by up to 1000‐fold higher, than in normal tissues.^71^ *S typhimurium* has been utilized as a carrier to transport various converting enzymes and antigens into tumors.^76^ The genus *Clostridium* is strictly anerobic and the vast majority of species can produce spores permitting survival but not growth in hypoxic conditions.^77^ Once conditions are favorable (as in wounds or spoiled meat), these spores can germinate into metabolically active bacterial cells. Furthermore, clostridia utilized in the field are generally susceptible to a wide range of antibiotics, permitting control of their replication at any time.^77^ Highly hypoxic tissue is usually only found in tumors and is absent in most other organs of the body. This high specificity was shown by Malmgren et al,^78^ who injected *Clostridium* spores into tumor‐bearing mice, and showed that only the mice with tumors died from the infection. In view of the fact that necrotic mainly areas exist only inside tumors and not in normal tissues, it was realized that lethal toxin‐free *Clostridium novyi* NT spores could be very efficient in eradicating established tumors.^66^, ^67^, ^79^ The systemic administration of clostridia spores (which is remarkably well tolerated) can lead to the destruction of tumor cells surrounding necrotic and hypoxic regions that are resistant to conventional therapies.^80^ The majority of anerobic bacteria tested up to now can form extremely resistant spores that enable them to survive even in oxygen‐rich conditions, but they cannot multiply or grow there. However, when they experience favorable conditions, for example, dead regions within tumors, the spores will germinate and the bacteria will multiply, rendering them ideal to target cancers. Spores of *C novyi*‐NT (a genetically modified strain without the lethal toxin) have demonstrated antitumor activity without any systemic side effects. In mice receiving an intratumoral injection of *C histolyticum* spores, a marked lysis of tumor tissues was observed. A similar phenomenon was found in mice injected intravenously with spores of *C sporogenes*. Additionally, *Clostridium* was not detected in normal tissues of mice receiving an intravenous injection of bacteria, but only in tumors. Pharmacological and toxicological assessment of mice injected with *C novyi*‐NT spores suggested that the spores were quickly cleared from the general circulation by the reticuloendothelial system. No systemic toxicity was seen in healthy mice or rabbits even after massive doses. However, in tumor‐bearing mice, toxicity appeared to be associated with the tumor size, and in this case, spores did cause bacterial infection. Bacterial spores have been additionally exploited as delivery agents for cytotoxic peptides, anticancer agents, therapeutic proteins, and as vectors for gene therapy.^6^, ^40^, ^47^

## 7 COMBINATION OF BACTERIOTHERAPY WITH DIFFERENT APPROACHES IN CANCER THERAPY

The combination of bacteriotherapy with other different types of cancer therapy has shown remarkable potential for both diagnostic and therapeutic applications. Chemotherapy, radiotherapy, and immunotherapy are accepted as the major conventional types of cancer treatment.^81^ However, conventional strategies can cause numerous complications in patients including induction of cancer cell resistance, systemic toxicity, and immune suppression, not to mention that they have failed to completely eradicate all the cancer cells in most cases. It is known to be challenging to effectively administer radiotherapy or chemotherapeutic agents to the hypoxic and acidic regions in tumors. Incomplete tumor targeting, inadequate tissue penetration, and limited cancer cell toxicity are limitations of most cytotoxic drugs.^82^ Therefore, viruses or other vectors have been used for selective tumor targeting and cancer therapy. About 30% of cancer‐related deaths are caused by failure of local tumor control, suggesting that improving local control has the potential to improve the survival of one‐third of all cancer patients. Several ways to improve local tumor control are currently under investigation. Promising strategies seem to be those that combine existing therapeutic modalities with new approaches, such as combining ionizing irradiation with gene therapy.^83^

## 8 COMBINATION OF BACTERIOTHERAPY WITH RADIOTHERAPY

Radiotherapy remains central among the most effective approaches to treat many different cancers. Even so, damage to normal tissues cannot be completely avoided, representing an important limitation for the efficacy of this cancer treatment approach.^84^ One of the fundamental explanations for the lack of efficacy of radiotherapy in some solid tumors is the presence of hypoxic (i.e. poorly vascularized) zones that are resistant to radiation. However, this limitation could be an advantage for other approaches, for example, the use of facultative or obligate anerobic bacteria.^62^ Therefore, the radiotherapeutic doses could be lowered to spare surrounding healthy tissues. Contrasted with another investigative approach, gene therapy, bacteriotherapy has the advantage of avoiding genetic modification of tumor cells, which is rather inefficient and not risk‐free. The combination of radiotherapy with bacteria is a novel active area of investigation. In spite of the fact that there have been few investigations using bacteria to promote radiotherapy, this field may still become a practical approach in clinical radiation oncology. It has been shown that genetically engineered *Salmonella* bacteria have the desired properties of an antitumor vector. They can selectively replicate within tumors, and can express effector genes such as the herpes simplex thymidine kinase. Salmonella that targets tumors from a distant inoculation site, can mediate tumor growth suppression.^85^ Lipid A‐altered *Salmonella* auxotrophs were developed that displayed attenuated toxicity in mice and swine. These mutants demonstrated considerably reduced induction of host TNF‐α (a key mediator in bacteria‐mediated tumor therapy), yet retained the capacity for tumor multiplication and growth suppression, achieving accumulation in tumors of 10^9^ colony forming units (cfu)/g of tumor, which was 1000 times higher than normal tissues in mice.^86^, ^87^ The outcome was followed for tumor growth and mouse survival. When the bacteriotherapy was combined with radiation treatment it produced additional antitumor effects. In dose‐response studies with increasing doses of radiation but single dosages of *Salmonella*, the 2 agents together caused synergistic suppression of tumor growth (greater than additivity).^88^ The attenuated *S typhimurium* strain ΔppGpp (guanosine 5'‐diphosphate‐3'‐diphosphate) could be used to kill tumor cells. It was engineered to carry a pBAD plasmid coding for cytotoxic protein cytolysis A (ClyA) and also for bacterial luciferase (Lux) to allow optical imaging.^46^ ClyA is a 34‐kDa pore‐forming hemolytic protein, which can be produced *S typhimurium* and *E coli* without posttranslational modification.^89^ The ClyA cytotoxicity toward macrophages and mammalian cells induced cell caspase‐mediated cell death. A serious drawback of radiotherapy is that it is not clear whether the radiation affects cytotoxin‐expressing *Salmonella* that has localized in the tumor. Liu et al^37^ reported that the combination of radiotherapy and bacterial therapy using engineered *S typhimurium* ∆ppGpp reduced tumor growth compared with bacterial therapy alone. In addition, the researchers recently showed that the agonist of toll‐like receptor 5 (TLR5), called bacterial flagellin, as well as CBLB502 (its pharmacologically optimized derivative) could protect primates and rodents from gastrointestinal and hematopoietic radiation syndromes induced by total body irradiation.^84^, ^90^ In another investigation, Platt et al^88^ showed that the combination of X‐rays with *Salmonella* had supra‐additive antitumor effects, with a larger slope of the dose‐response curve. They additionally proposed that at higher radiation doses (25‐50 Gy), the supra‐additive effect was not clear, as this would have needed a full fractional dose‐response. Felgner et al^40^ recently reported that pre‐exposure to therapeutic bacteria (*E coli* Symbioflor‐2 or *Salmonella* SL7207 vector strains) in naive mice and tumor bearing mice that were subsequently immunized, caused a significantly difference in the phenotype of the microenvironment of colonized tumors. Bettegowda et al^91^ used a combination of spores of *C novyi‐NT* together with radiation therapy to treat transplanted tumors in mice. Their results indicated that *C novyi‐NT* spores alone only had very minor therapeutic effects, while the combination resulted in long‐term remission in a significant fraction of animals. In their studies partial and complete responses were found with the combination of external beam therapy or brachytherapy, and a single dose of *C novyi‐NT*. Nevertheless, they recommended that the combination therapy using conventional doses of radiation could be toxic for organs like the liver. On the other hand, they suggested that a combination of radioactive iodine with *C novyi‐NT* might enable patients to be treated with lower doses of radiolabeled antibodies, subsequently limiting toxicity to normal tissues, for example, to bone marrow. Nuyts et al^32^ tested bacteria as a potential gene delivery agent and combined it with radiation therapy. They isolated 2 radiation‐inducible genes of the SOS repair system (*recA* and *recN* genes) in *Clostridium acetobutylicum* DSM792, and confirmed these genes could be activated at a radiation dose of 2 Gy. These results suggested that fractionated radiotherapy could trigger repeated gene induction in bacteria leading to enhanced and prolonged protein expression.^32^ They suggested that the radio‐responsive *recA* promoter could increase TNFa production in recombinant clostridia after 2 Gy of irradiation.^83^ Jiang et al^10^ assessed the antitumor effects of combining RT with bacteria. In this investigation, *E coli* carrying pAClyA was injected into CT26‐bearing BALB/c mice, after which they were irradiated with different radiation doses (0, 8, 15, 21 Gy). This research showed that engineered bacteria such as *E coli* strain K‐12 can produce ClyA to enhance the therapeutic effects of radiation. Furthermore, their findings confirmed that radiotherapy and bacteriolytic therapy could inhibit the development of tumor metastasis. Table 2 summarizes the studies on the use of bacteria after or during radiotherapy as a combination cancer therapy.

**Table 2:** Summaries of studies on combination of bacteriotherapy and radiotherapy for cancer therapy

Strain(s)	Methods	Outcome	Ref
*Clostridium novyi‐NT*	Injection 3 × 10^8^ spores of *C novyi*‐NT and irradiation; external beam radiation (0.1 Gy/s, Cs‐137 source), systemic radioimmunotherapy with 500µCi of I‐131‐labeled T84.66 mAb and brachytherapy (10 Gy/day) used of plaques loaded with I‐125 seeds in different transplanted tumors in mice	*C novyi‐NT* plus external beam radiation led to tumor shrinkage in mice bearing HCT116 tumors	91
		*C novyi* with brachytherapy led to cure 100% of mice bearing HCT116 and HuCC‐T1 xenografts	
		Treated of the xenografts of colorectal cancer LS174T by combination of *C novyi‐NT* with radioimmunotherapy	
*C oncolyticum M55*	Injection *C. oncolyticum* M 55 spores with local tumor hyperthermy in mice bearing Ehrlich solid carcinoma, Harding‐Pasey‐melanoma and fibrosarcoma	Oncolysis of the tumors	163
*C oncolyticum M55*	IV injection *C. oncolyticum* M 55 spores in combination with local tumor hyperthermy 2305 NMRI‐mice bearing neck tumors	intensification of the oncolysis tumors after 12 h	164
*C oncolyticum M55*	local X‐irradiation and local HFH with iv spore‐ *C. oncolyticum M55* in Harding‐Passey‐Melanoma‐bearing mice	Relatively cure	165
*C acetobutylicum DSM792*	Induction of the recA and recN genes (involved in DNA repair) in *Clostridium* by radiation 2 Gy	Significant increase in b‐galactosidase activity	32
*C acetobutylicum DSM792*	Induction of the gene involved in recA gene in *Clostridium* by radiation 2 Gy	Significant increase TNFα	83
*E coli K‐12*	SC injection *E coli* containing expressing ClyA gene (5 × 10^7^CFU) with 21 Gy radiation murine CT26 colon carcinoma cells	Tumor shrinkage, suppressed metastatic tumor growth and prolonged the survival time	10
*Salmonella YS146 and YS166*	Combination treatment administration of X‐rays 5 to 15Gy with ip or iv injection *Salmonella* 2 × 10^5^ cfu into mice bearing B16F10 or Cloudman S91 melanomas	Suppression of tumors growth and prolonged mice survival	88
*Salmonella typhimurium SHJ2037*	Combination treatment radiotherapy 21Gy with iv injection *S typhimurium* (containing of Plasmid construction plasmid pBAD‐RLuc8‐clyA) into mice bearing colon tumor CT26	Regression of tumors	37

Several innovative approaches have been proposed to reduce radiotherapy‐induced normal tissue damage.^92^, ^93^ The application of bacteria could also be used to reduce normal tissue damage during or after RT. Certain strains of bacteria (particularly *Lactobacilli* and *Bifidobacteria*) have been proposed to reduce RT side effects.^25^, ^94^, ^95^, ^96^ The use of probiotics to preserve normal tissue during or after radiotherapy has also been shown in many clinical and preclinical studies.^97^, ^98^, ^99^, ^100^, ^101^, ^102^, ^103^, ^104^, ^105^, ^106^, ^107^, ^108^

## 9 COMBINATION OF BACTERIOTHERAPY WITH CHEMOTHERAPY

Chemotherapy is still the mainstay of treatment for inoperable cancer, despite numerous shortcomings such as inadequate drug concentrations in tumors, occurrence of systemic toxicity (hematological, gastrointestinal, alopecia, heart, and skin toxicity) in many types of cancer, and almost inevitable induction of drug resistance.^109^, ^110^, ^111^, ^112^ Neutropenia is one of the main manifestations of hematological toxicity. It is well known that due to immunosuppression, neutropenia poses a risk of infectious disease occurring during treatment. In addition, chemotherapy is responsible for gastrointestinal toxicity because of mucosal damage, and altering the natural host microflora.^113^, ^114^, ^115^ It should be noted that tumor cells that remain after chemotherapy frequently show increased aggressiveness, and can enter blood and lymph vessels, thereby increasing the probability of metastasis.^116^, ^117^ Therefore the introduction of new approaches is required to increase effectiveness and reduce toxicity in chemotherapy.^109^ Combining chemotherapy with bacteriotherapy could be one of these new approaches. It should be pointed out that bacteria can specifically target the most hypoxic tumors which are often those most resistant to chemotherapy.^118^ Bacteria can, not only sensitize tumors to increase the efficiency of chemotherapy, but they can also be exploited as drug/gene delivery vehicles. Bacterial toxins can destroy tumors and can also be used for bacterial‐based cancer vaccines.^6^, ^61^ Genetically modified bacteria can be used for selective tumor targeting as well as bacterial gene‐directed enzyme prodrug therapy for cancer.^6^ Moreover, bacterial endotoxins can also be used to fight cancer particularly in combination with chemotherapy.^7^ Probiotic bacteria could mitigate the severity of chemotherapy‐induced toxicity, particularly the gastrointestinal side‐effects. VSL‐3 is 1 type of probiotic formula that has been effective in reducing complications (such as diarrhea induced by chemotherapy in rats).^119^ Whitford et al, showed that *Streptococcus thermophilus* has beneficial effects on 5‐fluorouracil (5‐FU) complications (intestinal mucositis).^120^ Bowen et al suggested that, in spite of the overall shortage of hard data, probiotics should be tested for chemotherapy complications.^113^ In another investigation, 150 patients with colorectal cancer receiving 5‐FU and leucovorin bolus injections plus continuous 5‐FU infusion as postoperative adjuvant therapy, were randomly allocated to receive *L rhamnosus* GG (1‐2 × 10(10) CFU) and fiber (11 g guar gum) per day, while others did not. The patients receiving *Lactobacillus* had significantly less severe grades of diarrhea. These participants also had less abdominal discomfort thereby reducing the need for hospital care and lowering of chemotherapy doses.^121^ Abd El‐Atti et al showed the effectiveness of probiotics to control chemotherapy complications in patients with advanced breast cancer.^122^ One of the most troubling treatment‐related complications in patients with head and neck cancer is oral mucositis. Some of the recent studies have investigated the positive effects of *Lactobacillus brevis* CD2 lozenges on the severity and prevalence of mucositis, as well as the tumor resistance to radiotherapy. It was observed that patients who received *Lactobacillus* during chemotherapy had fewer intestinal problems than the others, resulting in shortening the course of chemotherapy and lower doses.^94^, ^102^, ^108^, ^112^, ^123^, ^124^, ^125^ Another method to reduce the side effects of chemotherapy is the COBALT strategy (combination bacteriolytic therapy; simultaneous use of *C novyi*‐*NT* spores with conventional chemotherapeutic agents). Although COBALT showed meaningful antitumor effects, it could not completely prevent animal deaths.^66^ Another use of *C novyi‐NT* is its membrane‐disrupting potential in liposome‐encapsulated drug delivery to tumor cells.^126^, ^127^ Nitroreductase (NR) enzymes from different bacterial strains have been investigated in some studies.^128^ It was shown that the NR enzyme from *Haemophilus influenza* had promising pharmacokinetic properties and could be utilized in treating tumors in mice. Mice were treated either with *C sporogenes* alone, or with *C sporogenes* in combination with NR and CB1954.

A novel study demonstrated the power of *botulinum* neurotoxin *(BoNT*) to destroy tumor vessels, allowing greater cancer cell destruction by chemotherapy.^129^ The most important problem in this process was insufficient tumor lysis. Since all the components of malignant tissue are not completely consumed by bacteria, bacteriotherapy should be combined with chemotherapy.^130^ *Salmonella* and *Clostridium* produce the suicide enzyme cytosine deaminase (CDase) which transforms the pro‐drug 5‐fluorocytosine (5‐FC) to chemotherapeutic 5‐FU.^42^, ^131^, ^132^, ^133^ Nemunaitis et al in an experimental clinical trial used 5‐FC and recombinant *Salmonella* expressing CDase, and 66.7% of patients showed a tumor response.^18^ Another study investigated the ability and efficiency of *Salmonella typhimurium VNP20009* in a murine melanoma model combined with different chemotherapy drugs.^118^ Kasinskas et al,^134^ discussed the relationship between *S typhimurium* and the microenvironments of solid tumors. They suggested that the interaction of *S typhimurium* with the microenvironment regulated the amount and location of bacterial accumulation. Through monitoring these interactions, they proposed that administration of *S typhimurium* could lead to increased effects of standard chemotherapeutic drugs. Exploiting *S choleraesuis* as a single tumor‐targeting anticancer agent in tumor‐bearing mice was reported by Lee et al.^135^ Their study indicated that the combination of *S choleraesuis* and cisplatin postponed tumor development and increased survival.

## 10 BACTERIA IN THERANOSTIC APPROACHES

Theranostics describes the use of multifunctional approaches to simultaneously image, monitor and treat tumors, and has recently attracted a great deal of attention.^136^, ^137^, ^138^ One of the common approaches to theranostics is to use drug‐delivery nanovesicles, that also incorporate an imaging component.^139^ Bacteria can be used in theranostics because they can specifically target tumors and can replicate in tumor cells.^6^, ^139^ The ease of genetic manipulation of bacteria allows for the production of attenuated strains with greater safety profiles, and vector systems, thereby allowing for precise tuning and multifunctional capabilities.^7^, ^140^ To facilitate the monitoring of migration patterns and to follow the proliferation of these bacteria, scientists use genetically modified bacteria to express reporter genes that allow optical imaging.^10^ In addition, bacteria can be detected by MRI or PET.^7^, ^10^, ^140^ Sheng‐Nan Jiang et al,^10^ used an *E coli* strain K‐12 (MG1655) producing the cytotoxic protein and pore‐forming hemolytic cytolysin A (to kill colon cancer cells) and the bacterial luciferase (*Lux*) operon (as an in vivo imaging marker). They found that a combination of bacteriotherapy and radiotherapy reduced tumor metastasis and increased the survival rate in mice. Cheng‐Hung Luo developed 2 approaches for cancer theranostics using *Bifidobacterium breve* and *Clostridium difficile* to increase the treatment and imaging effectiveness.^141^ In another study, Zurkiya et al^142^ applied magnetotactic bacteria (naturally producing magnetosomes) to deliver a gene expression marker *(magA*) in the human 293FT cell line as a candidate MRI reporter gene. Quispe‐Tintaya et al tested an engineered bacterial strain (virulence‐attenuated live *Listeria monocytogenes* with bound radioactive antibodies), instead of administering radiotherapy and bacterial tumor therapy independently. They observed that this construct led to primary tumor regression (>60%) and reduced metastases (>90%).^143^ Some bacteria (*Haloarchaea*) that produce gas vesicles (~width of 45‐250 nm and length of 100‐600 nm) have been examined as theranostic agents.^144^, ^145^ Recently, Shapiro et al^146^ described a novel diagnostic strategy using gas vesicles (harvested from *Anabaena flosaquae* and *Halobacterium* NRC‐1) as ultrasound contrast agents for molecular imaging in mice. In addition, they suggested that these vesicles could be targeted as therapeutic agents and drug or gene delivery vehicles.^147^

## 11 CONCLUSIONS AND PROSPECTIVES

The unique pathophysiology of solid tumors causes major obstacles for traditional anticancer therapies**.** There are advantages and disadvantages in the applications of therapeutic bacteria in cancer therapy.^18^, ^134^ Currently, although traditional cancer therapies are still the mainstream treatments, bacteriotherapy has demonstrated remarkable effects, thanks to its high specificity, ability to be controlled post‐administration, and oncolytic capabilities in many in vivo studies.^34^ Nevertheless, many problems remain for using bacteria in clinical practice as antitumor agents including; bacterial toxicity, DNA instability, limited targeting efficiency, choice of practical and safe bacterial strains, and testing combination with other therapies.^7^, ^80^, ^148^ Hopefully, these obstacles can be overcome by more sophisticated genetic engineering of tailored strains. In the future, genetically modified bacteria will be made more practical for both diagnostic and therapeutic anticancer applications, and to enhance radiotherapy, immunotherapy, or chemotherapy efficacy.

## CONFLICT INTEREST

There are no conflicts of interest.
